# Insights into polycrystalline microstructure of blood films with 3D Mueller matrix imaging approach

**DOI:** 10.1038/s41598-024-63816-z

**Published:** 2024-06-13

**Authors:** Alexander G. Ushenko, Anton Sdobnov, Irina V. Soltys, Yuriy A. Ushenko, Alexander V. Dubolazov, Valery M. Sklyarchuk, Alexander V. Olar, Liliya Trifonyuk, Alexander Doronin, Wenjun Yan, Alexander Bykov, Igor Meglinski

**Affiliations:** 1 Taizhou Institute of Zhejiang University, Taizhou, 310027 China; 2 Optics and Publishing Department, Yuriy Fedkovych Chernivtsi National University, 2 Kotsiubynskyi Str., Chernivtsi, 58002 Ukraine; 3 Optoelectronics and Measurement Techniques, University of Oulu, P.O. Box 4500, 900014 Oulu, Finland; 4 Department of Physics, Shaoxing University, Shaoxing, Zhejiang, 312000 China; 5 Computer Science Department, Yuriy Fedkovych Chernivtsi National University, 2 Kotsiubynskyi Str., Chernivtsi, 58002 Ukraine; 6 Rivne State Medical Center, 78 Kyivska Str., Rivne, 33007 Ukraine; 7 School of Engineering and Computer Science, Victoria University of Wellington, 6140 Wellington, New Zealand; 8 College of Engineering and Physical Sciences, Aston University, Birmingham, B4 7ET UK

**Keywords:** Liquid biopsy, Polarized light, 3D Mueller matrix, Blood, Polycrystalline thin films, Birefringence, Cancer diagnosis, Imaging and sensing, Optical sensors

## Abstract

This study introduces a novel approach in the realm of liquid biopsies, employing a 3D Mueller-matrix (MM) image reconstruction technique to analyze dehydrated blood smear polycrystalline structures. Our research centers on exploiting the unique optical anisotropy properties of blood proteins, which undergo structural alterations at the quaternary and tertiary levels in the early stages of diseases such as cancer. These alterations manifest as distinct patterns in the polycrystalline microstructure of dried blood droplets, offering a minimally invasive yet highly effective method for early disease detection. We utilized a groundbreaking 3D MM mapping technique, integrated with digital holographic reconstruction, to perform a detailed layer-by-layer analysis of partially depolarizing dry blood smears. This method allows us to extract critical optical anisotropy parameters, enabling the differentiation of blood films from healthy individuals and prostate cancer patients. Our technique uniquely combines polarization-holographic and differential MM methodologies to spatially characterize the 3D polycrystalline structures within blood films. A key advancement in our study is the quantitative evaluation of optical anisotropy maps using statistical moments (first to fourth orders) of linear and circular birefringence and dichroism distributions. This analysis provides a comprehensive characterization of the mean, variance, skewness, and kurtosis of these distributions, crucial for identifying significant differences between healthy and cancerous samples. Our findings demonstrate an exceptional accuracy rate of over $90\%$ for the early diagnosis and staging of cancer, surpassing existing screening methods. This high level of precision and the non-invasive nature of our technique mark a significant advancement in the field of liquid biopsies. It holds immense potential for revolutionizing cancer diagnosis, early detection, patient stratification, and monitoring, thereby greatly enhancing patient care and treatment outcomes. In conclusion, our study contributes a pioneering technique to the liquid biopsy domain, aligning with the ongoing quest for non-invasive, reliable, and efficient diagnostic methods. It opens new avenues for cancer diagnosis and monitoring, representing a substantial leap forward in personalized medicine and oncology.

## Introduction

Over the past decades, there have been extensive studies on the formation of complex patterns arising during the evaporation of liquid droplets ^1^. Distinct patterns, including coffee rings ^2^, cracking ^3^, and gelation ^4^, have been observed in biofluid droplets during drying. These patterns hold potential as straightforward diagnostic tools for assessing the health of both humans and livestock ^5,6^.

The dried blood droplet displays a discernible structure comprising three zones with varying thickness ^7^. Peripheral zone, characterized by a polycrystalline layer of albumin exhibiting linear birefringence and dichroism. Transitional zone, consisting of external and internal layers of optically isotropic cubic crystals of $Na-Cl$ salt, with an intermediate layer of globulin demonstrating circular birefringence and dichroism. Central zone, featuring an external layer of cubic crystals of $Na-Cl$ salt. All zones contain multiple-scattering optical radiation elements-erythrocytes, platelets, and leukocytes-with manifestations of circular birefringence and dichroism ^8^. Alterations in the cellular and macromolecular constituents of blood, induced by diseases, are believed to contribute to variations in the dried drop patterns of both plasma and whole blood ^5,6^.

Spectroscopic techniques, including Raman, surface-enhanced Raman spectroscopy (SERS), infrared (IR), Fourier Transform IR (FTIR), and vibrational spectroscopy, have demonstrated the ability to characterize biomolecular presence and generate a biochemical fingerprint, offering implications for indicating disease states through the detection of protein imbalances within the drop during liquid evaporation ^6^. Alternatively to the spectroscopic techniques, the study explores spatially non-uniform, optically anisotropic biological structures with multiple-scattering layers using the Mueller matrix (MM)-based polarimetry approach ^9–12^. This method extracts mediated information, represented by 16 MM elements, and integrates it to provide a comprehensive understanding of the polycrystalline structure within the biological layer, covering all scattering (depolarizing) inhomogeneities throughout the volume. The results obtained from MM microscopy are objectively assessed through statistical analysis of MM images and maps of optical anisotropy ^13,14^. This approach enabled the quantitative measurement of optical indicators to characterize the progression of gastric tissue from a healthy state through inflammation to cancer, utilizing Mueller microscopy of gastric biopsies ^13^. The shift from static configurations to the development of dynamic polarization systems represents a promising avenue for advancing MM microscopy. It has been demonstrated that incorporating ultrafast stereo polarimetric compressed photography (SP-CUP) into polarimetric systems can significantly enhance resolution by capturing high-dimensional events at the speed of light in a single exposure. It has been shown that one of the promising directions for increasing the resolution of polarimetric systems is the introduction of ultrafast stereo polarimetric compressed photography (SP-CUP) to record high-dimensional events at the speed of light in a single exposure. Combining compressed sensing and string imaging with stereoscopy and polarimetry, SP-CUP enables video recording of five photonic tags (*x*, *y*, *z*—space, *t*—time of arrival, and $\psi$—linear polarization angle) at 100 billion frames per second. second with picosecond time resolution. This capability has enabled the ultrafast three-dimensional imaging of the linear polarization properties of a single ultrashort laser pulse as it propagates through a scattering medium.

A further development of the Jones and Mueller matrix methods was the synthesis of the principles of interferometry and polarization mapping in the study of biological samples and environments. A notable application of this technique is detailed in ^15^, which introduces the fundamentals of a compact polarization linear holography system. This approach is capable of one-shot extraction of Jones matrix elements, utilizing a compact polarization geometry to simultaneously generate orthogonal polarization states and detect linear holograms with polarization multiplexing. The imaging compatibility and measurement accuracy of this method have been experimentally validated through real-time synthesis of Jones matrix elements, using both specially designed polarization-sensitive samples and a standard birefringence target.

As a result, polarization-sensitive optical coherence tomography (PS OCT) methods have been developed. Over the past 25 years, the principles and significant findings of PS OCT have been extensively detailed in a series of review articles ^16–18^. This polarization-interference technique enables the acquisition of layer-by-layer distributions of elements from the Jones and Mueller matrices within a shallow depth of up to 2 mm in biological tissues ^19–23^. Based on this imaging technique, accurate diagnoses of fibrosis and differentiation of tumor areas with weak fibrosis were possible ^24^. However, the resolution of these systems, ranging from 8 to 10 µm, and their sensitivity were constrained by the distorting effects of a high level of depolarized laser speckle background, which reduced the contrast of polarization images of tissue layers. Furthermore, PSOCT systems are costly and lack the capability to quantitatively analyze the optical parameters of biological tissues. Despite these limitations, these studies have unveiled new possibilities for three-dimensional polarimetric biomedical differential diagnosis in diffuse samples of benign and malignant tumors of human organs.

A significant target for advancing polarimetric biomedical optics is the refinement of polarization-interference techniques to mitigate the effects of depolarized backgrounds caused by multiple scattering in biological layers. It is crucial to enhance the detection and isolation of laser field components from objects with varying degrees of light scattering through digital holographic reconstruction and phase scanning, aiming for higher resolution between 2 and 3 micrometers ^9,10,12^. The integration of these optical experimental principles is expected to facilitate the extraction of MM images of single-scattering polycrystalline structures in biological objects. Such advancements could not only improve the sensitivity of PSOCT methods but also establish clear quantitative diagnostic correlations between the statistical parameters of MM images structures and the optically fine components of biological layers’ polycrystalline architectonics.

Despite the notable achievements of polarization and interference matrix techniques in analyzing the structure of biological tissues, their application in examining the polycrystalline structure of various biological fluids from human organs remains limited and insufficiently tested. Physiological and pathological processes in a living organism induce specific structural changes in proteins and other organic molecules. These molecular transformations could potentially provide a foundation for the early diagnosis of various diseases ^25–27^. Furthermore, a compelling practical advantage is that obtaining biological fluids is a minimally invasive and generally safe procedure, in contrast to the more traumatic and risky tissue biopsies. A key area of diagnostics involves the study of dehydrated films (facies) of biological fluids (BL). The structure of these facies provides insights into the composition and interrelationships of substances dissolved in the BL. Analysis of these systems at the macroscopic level of self-organization offers valuable information about their molecular structure ^28,29^.

Presently, only a quite limited number of polarization studies on facies of biological liquids (BL) such as blood plasma, urine, and others have been reported ^30–37^. These studies employ methods of two-dimensional (2D) polarization mapping to analyze microscopic images of various BL facies. For quantitative analysis, a range of algorithms including statistical, correlation, fractal, wavelet, and Fourier analysis are utilized. These analyses have led to the identification of initial objective markers that enable the differential diagnosis of various pathologies in fields such as urology, gynecology, and oncology. However, the further development of these methods has been hindered by the distorting effects of multiply scattered depolarized backgrounds on polarization images of facies and the absence of algorithms capable of digitally reconstructing optical anisotropy maps of supramolecular polycrystalline networks.

Current research is focused on advancing the technique of polarization-interference Mueller matrix layer-by-layer digital holographic reconstruction. Specifically, we aim to reconstruct depolarization maps of native histological sections of prostate tissue biopsies ^38^. The goal is to enhance the clinical utility of this method through the development of algorithms that can generate maps of optical anisotropy of supramolecular networks in polycrystalline blood facies. This advancement will facilitate the derivation of a set of statistical parameters (markers), which are crucial for accurately differentiating the stages of prostate cancer.

In contradistinction to conventional tissue specimens, anisotropic structures, exemplified by dry blood smears, offer a readily accessible alternative that eliminates the necessity for invasive biopsy procedures. The optical scrutiny of blood smears emerges as a promising and expeditious screening modality, particularly in the context of conditions such as prostate cancer, which instigates discernible alterations in the optical anisotropy characteristics ^9,12^. This MM polarimetry approach presents a non-traumatic and straightforward methodology for screening purposes.

**Figure 1:**
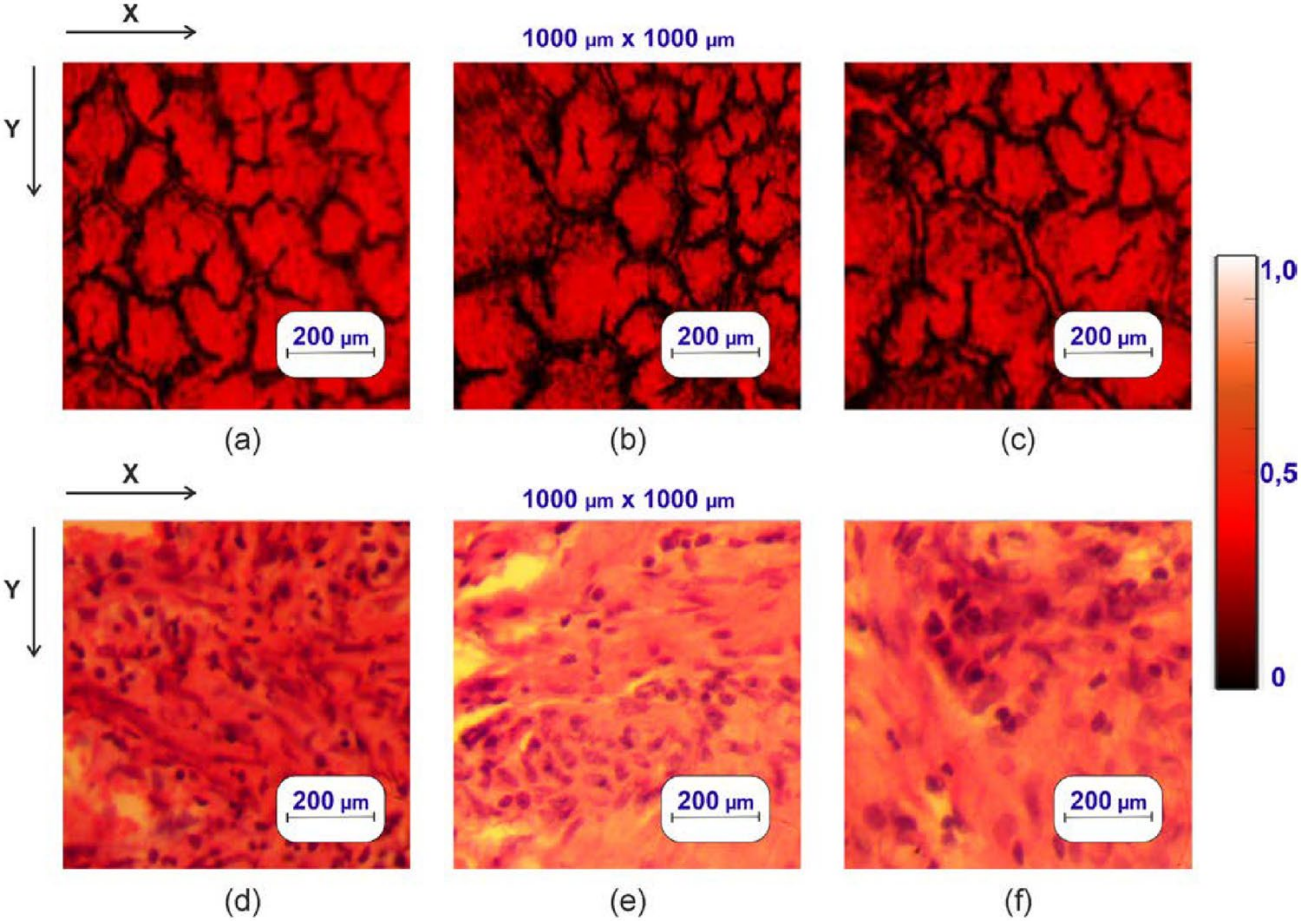
Microscopic and histological images of blood smears and prostate tissue biopsies—thin (2–5 µm) films, respectively: (**a**) and (**d**) show blood smears and prostate tissue at normal conditions; (**b**) and (**e**) depict high-differentiation adenocarcinoma conditions; (**c**) and (**f**) illustrate low-differentiation adenocarcinoma.

Figure 1a–v displays blood smears from a healthy donor (a) and patients with adenocarcinoma of high (see Fig. 1b) and low (see Fig. 1c) differentiation. Figure 1 shows histological and microscopic images of prostate tissue biopsies and blood smears. Figure 1d–f consists of histological preparations of healthy prostate tissue (see Fig. 1d) and adenocarcinoma of high (see Fig. 1e) and low (see Fig. 1f) differentiation.

For practical clinical applications, it is crucial to extend MM polarimetry diagnostic techniques to assess the 3D polycrystalline structures of biological layers characterized by varying light scattering multiplicities and distinctive depolarizing capabilities. The layer-by-layer mapping of individual elements within the MM, specifically characterizing the parameters of phase and amplitude anisotropy, holds the potential to furnish critical diagnostic information. Achieving this goal involves the integration of previously reported differential MM techniques ^39–44^ with holographic mapping of phase-inhomogeneous layers ^9,12^.

The layer-by-layer distributions of depolarization degree, when aggregated, offer a thorough three-dimensional representation of depolarization and individual anisotropy parameters at a local scale. Preliminary investigations utilizing this methodology have unveiled a correlation between tissue features and three-dimensional Mueller matrix (3D MM) imaging, as evidenced in prior research ^10^. This correlation establishes the groundwork for an effective and highly accurate differential diagnosis of prostate tumor tissues ^38^.

In current study, we introduce the 3D MM mapping technique employing digital holographic reconstruction for the layer-by-layer profiling of partially depolarizing dry blood smears—thin films. This technique facilitates the extraction of optical anisotropy parameters. Our results establish criteria for distinguishing polycrystalline blood films from those of healthy donors and patients with prostate cancer. Notably, through the integration of polarization-holographic and differential MM methodologies, we introduce, to the best of our knowledge, a novel approach for the spatial 3D characterization of polycrystalline structures within blood films.

### 3D Mueller matrix imaging approach: basic equations and theoretical remarks

Traditionally, samples containing spatially inhomogeneous optically anisotropic diffuse layers are studied using MM polarimetry approaches ^45–52^. In this approach, only indirect and averaged data (in the form of 16 matrix elements) can be obtained, representing the entire volume of scattering (depolarizing) inhomogeneities. To develop a new, more sensitive, and unambiguous method for tissue diagnosis, it is necessary to comprehensively address several theoretical and experimental challenges and synthesize the obtained results. The principles and steps of the proposed research are outlined in Table 1.

**Table 1:** Structural and logical scheme of the 3D layer-by-layer MM image reconstruction approach.

No.	Task	Method	Result
1	Extraction of direct information about the distribution of optical anisotropy parameters	Differential MM Mapping	Algorithms for the reconstruction of the values of linear and circular birefringence and dichroism averaged over the volume of a polycrystalline layer
2	Reducing the influence of the depolarized background	Polarization-holographic recording and restoration of the object field	Algorithms for layer-by-layer restoration of the amplitude-phase structure of the object field of a polycrystalline layer
3	Obtaining layer-by-layer distributions of optical anisotropy parameters of a polycrystalline layer	Phase scanning of the amplitude-phase structure of the object field	Layer-by-layer coordinate distributions of linear and circular birefringence and dichroism values
4	Synthesis of methods 1–3 for the polarization-holographic investigation of polycrystalline structure of blood films		
5	Statistical analysis of layer-by-layer maps of linear and circular birefringence and dichroism of polycrystalline structure of blood films		
6	Data analysis and determination of the diagnostic power (sensitivity, specificity, accuracy) of early diagnosis of prostate cancer		

#### Differential Mueller matrix mapping

The theoretical foundations of the Mueller MM approach, which describes the interaction of optical radiation with depolarizing layers, are comprehensively detailed in numerous studies ^16–23^. Specifically, under conditions of multiple scattering, the MM of a depolarizing layer exhibits variation along the direction of light propagation. This relationship is analytically represented by the following equation:

1 $$\begin{aligned} d\frac{\{R\}\{z\}}{dz}=\{R\}\{z\}\{W\}(z), \end{aligned}$$

where $\{R\}(z)$ is the MM of an object in a plane at *z* in the direction of propagation, and $\{W\}(z)$ is the corresponding differential MM. For optically thin, non-depolarizing layers, the differential matrix *W*(*z*) incorporates six elementary polarization properties, collectively providing a complete characterization of the optical anisotropy of the biological layer

2 $$\begin{aligned} \big \{W\big \}(z)=\left\| \begin{array}{cccc} 0 &{} LD &{} LD^* &{} CD \\ LD &{} 0 &{} CB &{} -LB^* \\ LD' &{} -CB &{} 0 &{} LB \\ CD &{} LB' &{} -LB &{} 0 \end{array}\right\| (z). \end{aligned}$$

Here, *LD* and *LB* are the linear dichroism and birefringence for a direction of the optical axis $y=0^{\circ }$; $LD^*$ and $LB^*$ are the linear dichroism and birefringence for a direction of the optical axis $y=45^{\circ }$; and *CD* and *CB* are the circular dichroism and birefringence determined by the following relations $LB=\frac{2\pi }{\lambda } {\Delta }n_{LB}l$; $LB^*=\frac{2\pi }{\lambda } {\Delta }n_{LB}^*l$; $CB=\frac{2\pi }{\lambda } {\Delta }n_{CB}l$; $LD= {\Delta }\tau _{\left( 0^0-90^0\right) }=tg\gamma$; $LD^*= {\Delta }\tau _{\left( 45^0-135^0\right) }=tg\left( \gamma +45^0\right)$; $CD=\frac{\chi _\otimes -\chi _\oplus }{\chi _\otimes +\chi _\oplus }$. For a diffuse medium, the matrix 2 can be represented as separate average $\langle \{W\}\rangle (z)$ (polarisation part) and fluctuating $\langle \{\tilde{W}\}\rangle (z)$ (depolarisation part) components

3 $$\begin{aligned} \{W\}(z)=\langle \{W\}\rangle (z)+\langle \{\tilde{W}\}\rangle (z). \end{aligned}$$

It should be noted that feedback is always present as

4 $$\begin{aligned} \left\{ R\right\} \left( z\right) =exp\left[ \left\{ W\right\} \left( z\right) \right] . \end{aligned}$$

Common analysis of (1-4) facilitated the derivation of an expression for the logarithmic matrix algorithm (LMA)

5 $$\begin{aligned} LMA\left( z\right) =ln\left[ \left\{ R\right\} \left( z\right) \right] ={LMA}_{Pol}+{LMA}_{Dep} \end{aligned}$$

that is determined by a superposition of antisymmetric polarization components ${LMA}_{Pol}$ and symmetric depolarization) components ${LMA}_{Dep}$ of $LMA\left( z\right)$

6 $$\begin{aligned} \left\{ \begin{array}{llll} {LMA}_{Pol}=\langle \{W\}(z)\rangle \times z=0.5(LMA(z)-GLMA(z)^TG);\\ {LMA}_{Dep}=\langle \{W\}(z)\rangle \times z=0.5(LMA(z)+GLMA(z)^TG);\\ G=diag\left( 1;-1;-1;-1\right) ,\\ \end{array}\right. \end{aligned}$$

where G is the metric Minkovsky matrix ^41,44^.

Building on this foundation, this research utilized a model of generalized phase and amplitude anisotropy of a partial biological layer to develop and substantiate the principles of two-dimensional (2D) MM tomography. This was achieved through Stokes-polarimetry mapping of polycrystalline networks in the sample, followed by acquiring a series of Mueller-matrix images. These images were differentiated into fully polarized and depolarized components, culminating in the formulation of a set of algorithms ($0\le z\le l$) for layered reconstruction. These algorithms systematically reconstructed the distributions of mean values and fluctuations of parameters such as linear and circular dichroism and birefringence. Given the relations (2) and (4)–(6), the polarization component of the matrix logarithmic algorithm is expressed as follows:

7 $$\begin{aligned} \langle \big \{W\big \}\rangle =0.5z^{-1}\left\| \begin{array}{cccc} 0 &{} \left( {LMA}_{12}+{LMA}_{21}\right) &{} \left( {LMA}_{13}+{LMA}_{31}\right) &{} \left( {LMA}_{14}+{LMA}_{41}\right) \\ \left( {LMA}_{21}+{LMA}_{12}\right) &{} 0 &{} \left( {LMA}_{23}-{LMA}_{32}\right) &{} \left( {LMA}_{24}-{LMA}_{42}\right) \\ \left( {LMA}_{31}+{LMA}_{13}\right) &{} \left( {LMA}_{32}-{LMA}_{23}\right) &{} 0 &{} \left( {LMA}_{34}-{LMA}_{43}\right) \\ \left( {LMA}_{41}+{LMA}_{14}\right) &{} \left( {LMA}_{42}-{LMA}_{24}\right) &{} \left( {LMA}_{43}-{LMA}_{34}\right) &{} 0 \end{array}\right\| , \end{aligned}$$

where

8 $$\begin{aligned} \left\{ \begin{array}{llll} {LMA}_{ik}=ln{r_{ik}};\\ {LMA}_{ik}+{LMA}_{ki}=ln{\left( r_{ik}\times r_{ki}\right) };\\ {LMA}_{ik}-{LMA}_{ki}=ln{\left( \frac{M_{ik}}{M_{ki}}\right) }.\\ \end{array}\right. \end{aligned}$$

Taking into account relation 8, expression 7 can be rewritten as follows

9 $$\begin{aligned} \langle \big \{W\big \}\rangle =0.5z^{-1}\left\| \begin{array}{cccc} 0 &{} ln{\left( r_{12}r_{21}\right) } &{} ln{\left( r_{13}r_{31}\right) } &{} ln{\left( r_{14}r_{41}\right) } \\ ln{\left( r_{12}r_{21}\right) } &{} 0 &{} ln{\left( \frac{r_{23}}{r_{32}}\right) } &{} ln{\left( \frac{r_{24}}{r_{42}}\right) } \\ ln{\left( r_{13}r_{31}\right) } &{} ln{\left( \frac{r_{32}}{r_{23}}\right) } &{} 0 &{} ln{\left( \frac{r_{34}}{r_{43}}\right) } \\ ln{\left( r_{14}r_{41}\right) } &{} ln{\left( \frac{r_{42}}{r_{24}}\right) } &{} ln{\left( \frac{r_{43}}{r_{34}}\right) } &{} 0 \end{array}\right\| . \end{aligned}$$

The common analysis of relations 3–9 allows obtaining the algorithms of polarization-phase tomography—analytical expressions of Mueller-matrix layer-by-layer $(0\le z\le l)$ with the step ${\Delta z}$ reproduction of mean values of the parameters of phase and amplitude anisotropy of polycrystalline structure of optically thick biological layer

By collectively examining equations (2) and (4), algorithms can be deduced for reproducing the average values of the phase and amplitude anisotropy parameters:

10 $$\begin{aligned} \delta =\frac{2\pi z}{\lambda }\Delta n_{LB}=ln(\frac{r_{34}}{r_{42}}); \end{aligned}$$

11 $$\begin{aligned} \delta ^*=\frac{2\pi z}{\lambda }\Delta n^{*}_{LB}=ln(\frac{r_{24}}{r_{42}}); \end{aligned}$$

12 $$\begin{aligned} \phi =\frac{2\pi z}{\lambda }\Delta n^{*}_{CB}=ln(\frac{r_{23}}{r_{32}}); \end{aligned}$$

13 $$\begin{aligned} \Delta \tau _{(0^{\circ }-90^{\circ })}=tgy=ln(r_{12}r_{21}); \end{aligned}$$

14 $$\begin{aligned} \Delta \tau _{(45^{\circ }-135^{\circ })}=tg(y+45^{\circ })=ln(r_{13}r_{31}); \end{aligned}$$

15 $$\begin{aligned} \Delta \chi =\frac{\chi _{\otimes }-\chi _{\oplus }}{\chi _{\otimes }+\chi _{\oplus }}=ln(r_{14}r_{41}); \end{aligned}$$

where $\delta$ and $\delta ^*$ are the phase shifts between orthogonally polarised ($0^{\circ }{-}90^{\circ }$ and $45^{\circ }{-}135^{\circ }$) components of the amplitude of incident light; $\Delta n_{LB}$ and $\Delta n_{LB}^{*}$ are the magnitudes of the linear birefringence for $0^{\circ }{-}90^{\circ }$ and $45^{\circ }{-}135^{\circ }$ respectively; $\phi$ is the phase shift between the right- ($\otimes$) and left- ($\oplus$) circularly polarised components of the amplitude of laser radiation; $\Delta n_{CB}$ is the circular birefringence; $\Delta \tau _{(0^{\circ }{-}90^{\circ })}$and $\Delta \tau _{(45^{\circ }{-}135^{\circ })}$ are the ratios between the absorption coefficients of orthogonally polarised ($0^{\circ }{-}90^{\circ }$ and $45^{\circ }{-}135^{\circ }$) components of the amplitude of laser radiation; $\chi _{\otimes }$ and $\chi _{\oplus }$ are, respectively, the absorption coefficients of the right and left circularly polarised components of the laser radiation amplitude; and $\lambda$ is the laser wavelength.

Therefore, using the ideologies of differential data analysis of Mueller-matrix mapping allowed us to obtain a set of algorithms (Eqs. 10–15) of polarization reproduction of mean values of the parameters of phase and amplitude anisotropy of a polycrystalline component of biological layer.

#### Polarization-holographic recording and restoration of the object field

To determine the layer-by-layer distributions of matrix elements $r_{ik}$ six distinct polarization states are formed in the illuminating (*Ir*) and reference (*Re*) beams $(\{Ir-Re\}\Rightarrow 0^{\circ }; 90^{\circ }; 45^{\circ }; 135^{\circ }; \otimes ; \oplus$). For each polarization state (*p* *i* *r*), two partial interference patterns are registered through a polarizer-analyzer oriented at $\Omega =0^{\circ };\Omega =90^{\circ }$. For each partial interference distribution, two-dimensional discrete Fourier transform *F*(*u*, *v*) is further performed. The *F*(*u*, *v*) of a two-dimensional array $I_{\Omega =0^{\circ };90^{\circ })}(m,n)$ (the obtained image) is a function of two discrete variables coordinates (*m*, *n*) camera pixels defined by ^10^:

16 $$\begin{aligned} {\begin{matrix} \displaystyle &{}\Phi F_{x;y}(\Omega =0^{\circ };90^{\circ })(u,v)\\ &{}\quad =\frac{1}{M\times N}\sum ^{M-1}_{m=0}\sum ^{N-1}_{n=0} I_{x,y}(\Omega =0^{\circ };90^{\circ })(m,n)\\ &{}\quad \times \Bigl [-i2\pi \Bigl (\frac{m\times u}{M}+\frac{n\times v}{N}\Bigr ) \Bigr ], \end{matrix}} \end{aligned}$$

where

17 $$\begin{aligned} { \left\{ \begin{array}{llll} &{}I_{x}^{0}(\Omega =0^{\circ })(m,n)\\ &{}\quad =U_{x}^{0}(\Omega =0^{\circ })(m,n)(U_{x}^{0})^{*}(\Omega =0^{\circ })(m,n); \\ &{}I_{x}^{90}(\Omega =90^{\circ })(m,n)\\ &{}\quad =U_{x}^{90}(\Omega =90^{\circ })(m,n)(U_{x}^{90})^{*}(\Omega =90^{\circ })(m,n); \\ &{}I_{y}^{0}(\Omega =0^{\circ })(m,n)\\ &{}\quad =U_{y}^{0}(\Omega =0^{\circ })(m,n)(U_{y}^{0})^{*}(\Omega =0^{\circ })(m,n); \\ &{}I_{y}^{90}(\Omega =90^{\circ })(m,n)\\ &{}\quad =U_{y}^{90}(\Omega =90^{\circ })(m,n)(U_{y}^{90})^{*}(\Omega =90^{\circ })(m,n); \end{array}\right. } \end{aligned}$$

are the coordinate distributions of the intensity of the interference pattern filtered by the analyser with the orientation of its transmission axis at $\Omega =0^{\circ };\Omega =90^{\circ };$ ^∗^ denotes the complex conjugation operation; (*u*, *v*) are the spatial frequencies in the x and y directions respectively; and (*M*, *N*) are the number of pixels of the CCD camera in the m and n directions respectively, such that $0\le m$,$u \le M$ and $0\le n$,$v \le N$. The subsequent application of the two-dimensional inverse discrete Fourier transform on the obtained spectrum can be expressed as

18 $$\begin{aligned} {\begin{matrix} \displaystyle &{}\bigl [(\Phi T^{*}) \bigr ]_{\Omega =0^{\circ };90^{\circ }}^{0^{\circ };90^{\circ };45^{\circ };135^{\circ };\otimes ;\oplus }(u,v) \\ &{}\quad = \frac{1}{M\times N}\sum _{m=0}^{M-1}\sum _{n=0}^{N-1}I_{\Omega =0^{\circ };90^{\circ }}^{0^{\circ };90^{\circ };45^{\circ };135^{\circ }; \otimes ;\oplus }(m,n)\\ &{}\quad \times \Bigl [-i2\pi \Bigl (\frac{m\times u}{M}+\frac{n\times v}{N}\Bigr ) \Bigr ]. \end{matrix}} \end{aligned}$$

Here,

19 $$\begin{aligned} {\begin{matrix} &{}\bigl [(\Phi T^{*}) \bigr ]_{\Omega =0^{\circ };90^{\circ }}^{0^{\circ };90^{\circ };45^{\circ };135^{\circ };\otimes ;\oplus }(m,n)^{*}(x,y)\\ &{}\quad \equiv U_{\Omega =0^{\circ };90^{\circ }}^{0^{\circ };90^{\circ };45^{\circ };135^{\circ };\otimes ;\oplus }(m,n). \end{matrix}} \end{aligned}$$

Ultimately, the complex amplitude distribution for each polarization state can be derived in various phase planes $\theta _{k}=(\delta _{y}-\delta _{x})$ of the object field, separated by an arbitrary step of $\Delta \theta$:

20 $$\begin{aligned} \left\{ \begin{array}{lrlr} \Omega _{0^{\circ }}\rightarrow |U_{x}^{0}(\Omega =0^{\circ })|; \\ \Omega _{90^{\circ }}\rightarrow |U_{x}^{90}(\Omega =90^{\circ })|exp(i(\delta _{x}^{90}-\delta _{x}^{0})), \end{array}\right. \end{aligned}$$

21 $$\begin{aligned} \left\{ \begin{array}{lrlr} \Omega _{0^{\circ }}\rightarrow |U_{y}^{0}(\Omega =0^{\circ })|; \\ \Omega _{90^{\circ }}\rightarrow |U_{y}^{90}(\Omega =90^{\circ })|exp(i(\delta _{y}^{90}-\delta _{y}^{0})), \end{array}\right. \end{aligned}$$

#### Phase scanning of the amplitude-phase structure of the object field

The algorithm, described by (20) and (21), for scanning the phase of the complex amplitude field (18) and (19) directly corresponds to the physical depth $h_i$ of an optically anisotropic biological layer in the case of single scattering:

22 $$\begin{aligned} h_i=\frac{\lambda }{2\pi \Delta n}\theta _i. \end{aligned}$$

In the case of multiple scattering, the physical depth is multiplied (effective optical depth $h_{i}^{*}$) and becomes a multiple of the geometric thickness value of the biological layer *z*.

23 $$\begin{aligned} h_{i}^{*}\sim Kz. \end{aligned}$$

In each phase plane $\theta _k$ the corresponding parameters of the Stokes vector and polarization parameters of the object field of the biological layer are calculated as:

24 $$\begin{aligned} { \left\{ \begin{array}{llll} &{}ST_{1}^{0^{\circ };90^{\circ };45^{\circ };135^{\circ };\otimes ;\oplus }(\theta _k,m,n)\\ &{}\quad =(|U_0|^2+|U_{90}|^2)(\theta _k,m,n); \\ &{}ST_{2}^{0^{\circ };90^{\circ };45^{\circ };135^{\circ };\otimes ;\oplus }(\theta _k,m,n)\\ &{}\quad =(|U_0|^2-|U_{90}|^2)(\theta _k,m,n); \\ &{}ST_{3}^{0^{\circ };90^{\circ };45^{\circ };135^{\circ };\otimes ;\oplus }(\theta _k,m,n)\\ &{}\quad =2Re(U_{0}U_{90}^*)(\theta _k,m,n); \\ &{}ST_{3}^{0^{\circ };90^{\circ };45^{\circ };135^{\circ };\otimes ;\oplus }(\theta _k,m,n)\\ &{}\quad =2Im(U_{0}U_{90}^*)(\theta _k,m,n). \end{array}\right. } \end{aligned}$$

Based on relations (24), the elements of the MM $\{R\}$ is calculated using the following Stokes-polarimetric relations:

25 $$\begin{aligned} {\begin{matrix} &{}\big \{R\big \}(\theta _k,m,n)=\left\| \begin{array}{cccc} r_{11} &{}\quad r_{12} &{}\quad r_{13}&{}\quad r_{14} \\ r_{21} &{}\quad r_{22} &{}\quad r_{23} &{}\quad r_{24} \\ r_{31} &{}\quad r_{32} &{}\quad r_{33} &{}\quad r_{34} \\ r_{41} &{}\quad r_{42} &{}\quad r_{43} &{}\quad r_{44} \end{array}\right\| =0.5(\theta _k,m,n)\\ &{}\quad \times \left\| \begin{array}{cccc} (ST_{1}^{0}+ST_{1}^{90}) &{} (ST_{1}^{0}-ST_{1}^{90}) &{} (ST_{1}^{45}-ST_{1}^{135}) &{} (ST_{1}^{\otimes }-ST_{1}^{\oplus }) \\ (ST_{2}^{0}+ST_{2}^{90}) &{} (ST_{2}^{0}-ST_{2}^{90}) &{} (ST_{2}^{45}-ST_{2}^{135}) &{} (ST_{2}^{\otimes }-ST_{2}^{\oplus }) \\ (ST_{3}^{0}+ST_{3}^{90}) &{} (ST_{3}^{0}-ST_{3}^{90}) &{} (ST_{3}^{45}-ST_{3}^{135}) &{} (ST_{3}^{\otimes }-ST_{3}^{\oplus }) \\ (ST_{4}^{0}+ST_{4}^{90}) &{} (ST_{4}^{0}-ST_{4}^{90}) &{} (ST_{4}^{45}-ST_{4}^{135}) &{} (ST_{4}^{\otimes }-ST_{4}^{\oplus }) \end{array}\right\| . \end{matrix}} \end{aligned}$$

Using (4)–(10) the layer-by-layer distributions of the mean values of linear and circular birefringence and dichroism ($G(\langle LB \rangle ,\langle LB' \rangle , \langle CB \rangle , \langle LD \, \langle LD' \rangle , \langle CD \rangle )$) can be obtained :

26 $$\begin{aligned} \langle \ LB\rangle (\theta _k,m,n)=ln\Bigl (\frac{(ST_{3}^{\otimes }-ST_{3}^{\oplus })}{(ST_{4}^{45}-ST_{4}^{135})}\Bigr )(\theta _k,m,n); \end{aligned}$$

27 $$\begin{aligned} \langle \ LB'\rangle (\theta _k,m,n)=ln\Bigl (\frac{(ST_{2}^{\otimes }-ST_{2}^{\oplus })}{(ST_{4}^{0}-ST_{4}^{90})}\Bigr )(\theta _k,m,n); \end{aligned}$$

28 $$\begin{aligned} \langle \ CB\rangle (\theta _k,m,n)=ln\Bigl (\frac{(ST_{2}^{45}-ST_{2}^{135})}{(ST_{3}^{0}-ST_{3}^{90})}\Bigr )(\theta _k,m,n); \end{aligned}$$

29 $$\begin{aligned} {\begin{matrix} &{}\langle \ LD\rangle (\theta _k,m,n)\\ &{}=ln((ST_{1}^{0}-ST_{1}^{90})(ST_{2}^{0}+ST_{2}^{90}))(\theta _k,m,n); \end{matrix}} \end{aligned}$$

30 $$\begin{aligned} {\begin{matrix} &{}\langle \ LD'\rangle (\theta _k,m,n)=tg(y+45^{\circ })\\ &{}=ln((ST_{1}^{45}-ST_{1}^{135})(ST_{3}^{0}+ST_{3}^{90}))(\theta _k,m,n); \end{matrix}} \end{aligned}$$

31 $$\begin{aligned} {\begin{matrix} &{}\langle \ CD\rangle (\theta _k,m,n)\\ &{}=ln((ST_{1}^{\otimes }-ST_{1}^{\oplus })(ST_{4}^{0}+ST_{4}^{90}))(\theta _k,m,n); \end{matrix}} \end{aligned}$$

According to ^39–44^, we will further operate with the generalized quantities of linear birefringence and dichroism:

32 $$\begin{aligned} LB\equiv \sqrt{\langle \ CB\rangle ^2+\langle \ CB'\rangle ^2}; \end{aligned}$$

33 $$\begin{aligned} LD\equiv \sqrt{\langle \ LD\rangle ^2+\langle \ CD\rangle ^2}; \end{aligned}$$

In this manner, the synthesis of the 1st order differential matrix (1)–(10) and the algorithms for layer-by-layer polarization-holographic determination of matrix elements (16)–(25) allows the acquisition of layer-by-layer maps of linear and circular birefringence and dichroism of the polycrystalline structure of the dehydrated blood films (26)–(33).

## Results

**Figure 2:**
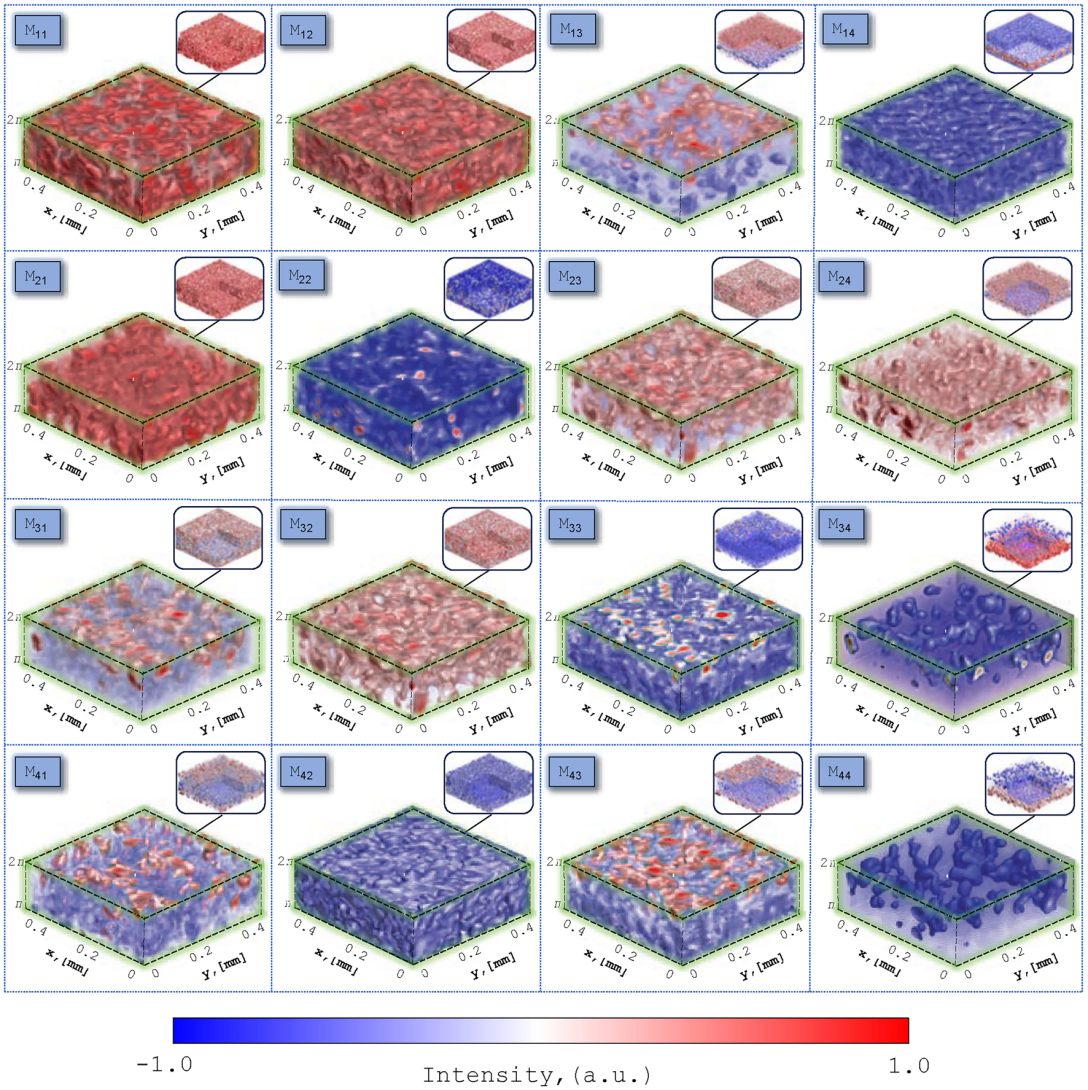
An example of typical polarization-interference measurement through digital holographic reconstruction, showcasing layered 3D elements of the MM for a dehydrated blood film. See Figure S1 in Supplementary Information for enlarged version of MM elements.

Figure 2 illustrates an example of polarization-interference measurement using digital holographic reconstruction of layered 3D elements of the MM of a dehydrated blood film. Each 3D MM element comprises a set of 200 phase-resolved 2D layers (ranging from 0 to $2\pi$ with a scanning step of $0.01\pi$). For each phase-resolved layer, the complex amplitude field (21) was reconstructed using algorithm (15). Subsequently, in this phase plane, the coordinate distributions of the MM elements are computed utilizing (25) and (26). Thus, through phase scanning of 3D MM elements, optical anisotropy maps (corresponding to Eqs. (5)–(10)) are reconstructed in each phase plane.

In current study, three phase slices at 0.2, 0.6, and 1.0 radians were utilized. The provided example illustrates the presence of all types of optical anisotropy mechanisms in the dehydrated blood film, as evidenced by the asymmetry in experimentally measured matrix elements. This observation can be theoretically explained by considering the presence of a complex mechanism involving linear birefringence and linear dichroism. The optical manifestations of linear birefringence in this mechanism are characterized by the partial MM:

34 $$\begin{aligned} \big \{D\big \}=\left\| \begin{array}{cccc} 1 &{}\quad 0 &{}\quad 0&{}\quad 0 \\ 0 &{}\quad d_{22} &{}\quad d_{23} &{}\quad d_{24} \\ 0 &{}\quad d_{32} &{}\quad d_{33} &{}\quad d_{34} \\ 0 &{}\quad d_{42} &{}\quad d_{43} &{}\quad d_{44} \end{array}\right\| , \end{aligned}$$

where

35 $$\begin{aligned} \left\{ \begin{array}{lrlr} d_{22}=cos^2 2\rho + sin^2 2\rho \, cos \, \delta , \\ d_{23}=d_{32}=cos \,2\rho \, sin \,2 \rho (1- cos \, \delta ), \\ d_{33}=sin^2 2\rho + cos^2 2 \rho \, cos \, \delta , \\ d_{42}=-d_{24}=sin \,2\rho \, sin \, \delta , \\ d_{34}=-d_{43}=cos \,2\rho \, sin \, \delta , \\ d_{44}=cos \, \delta . \end{array}\right. \end{aligned}$$

Here, $\rho$ is the optical axis direction, determined by the orientation of the polypeptide chain of amino acids; $\delta =\frac{2\pi }{\lambda } {\Delta nl}$ is the phase shift between linearly orthogonal polarized components of the laser beam amplitude; $\lambda$ is the wavelength, ${\Delta n}$ is the magnitude of birefringence; *l* represents the geometric thickness of the layer.

*Linear Dichroism* The analytical expression for the partial matrix operator that characterizes linear dichroism in optically anisotropic absorption is as follows:

36 $$\begin{aligned} \big \{\Psi \big \}=\left\| \begin{array}{cccc} 1 &{}\quad \phi _{12} &{}\quad \phi _{13}&{}\quad 0 \\ \phi _{21}&{}\quad \phi _{22} &{}\quad \phi _{23} &{}\quad 0 \\ \phi _{31}&{}\quad \phi _{32} &{}\quad \phi _{33} &{}\quad 0 \\ 0 &{}\quad 0 &{}\quad 0 &{}\quad \phi _{44} \end{array}\right\| , \end{aligned}$$

where

37 $$\begin{aligned} \left\{ \begin{array}{lrlr} \phi _{12}=\phi _{21}=(1-\Delta \tau )cos\, 2\rho , \\ \phi _{13}=\phi _{31}=(1-\Delta \tau )sin\, 2\rho , \\ \phi _{22}=(1+\Delta \tau )cos^2\, 2\rho +2\sqrt{\Delta \tau } sin^2\, 2\rho , \\ \phi _{23}=\phi _{32}=(1-\sqrt{\Delta \tau })^2 cos\, 2\rho \, sin\, 2\rho , \\ \phi _{33}=(1+\Delta \tau )sin^2\, 2\rho +2\sqrt{\Delta \tau } cos^2\, 2\rho , \\ \phi _{44}=2\sqrt{\Delta \tau }. \end{array}\right. \end{aligned}$$

Here, ${\Delta \tau }=\frac{\tau _x}{\tau _y}$, $$\bigl \{\begin{array}{lrlr} \tau _x=\tau \,cos\,\rho ;\\ \tau _y=\tau \,sin\,\rho , \end{array}$$ and $\tau _{x}$, $\tau _{y}$ is the coefficients of absorption for linearly polarized orthogonal components of laser radiation amplitude.

The resulting operator of two optical anisotropy mechanisms:

38 $$\begin{aligned} \big \{F\big \}=\left\| \begin{array}{cccc} 1 &{}\quad 0 &{}\quad 0&{}\quad 0 \\ 0 &{}\quad d_{22} &{}\quad d_{23} &{}\quad d_{24} \\ 0 &{}\quad d_{32} &{}\quad d_{33} &{}\quad d_{34} \\ 0 &{}\quad d_{42} &{}\quad d_{43} &{}\quad d_{44} \end{array}\right\| \times \left\| \begin{array}{cccc} 1 &{}\quad \phi _{12} &{}\quad \phi _{13}&{}\quad 0 \\ \phi _{21}&{}\quad \phi _{22} &{}\quad \phi _{23} &{}\quad 0 \\ \phi _{31}&{}\quad \phi _{32} &{}\quad \phi _{33} &{}\quad 0 \\ 0 &{}\quad 0 &{}\quad 0 &{}\quad \phi _{44} \end{array}\right\| , \end{aligned}$$

Observably, the symmetry of the birefringence matrix operator ($d_{23}=d_{32}$; $d_{34}=-d_{43}$;$d_{24}=-d_{42}$) is disrupted.

39 $$\begin{aligned} \begin{array}{c} f_{34}\ne f_{43};\\ f_{34}=d_{34}\phi _{44};\\ f_{43}=d_{42}\phi _{23}+d_{43}\phi _{33};\\ f_{23}\ne f_{32};\\ f_{23}=d_{22}\phi _{13}+d_{23}\phi _{22};\\ f_{32}=d_{32}\phi _{12}+d_{33}\phi _{32}. \end{array} \end{aligned}$$

### Layer-by-layer phase and amplitude anisotropy mapping in polycrystalline blood films: insights from healthy donors

By employing phase scanning techniques (20) and (21) on the reconstructed object field of complex amplitudes, we extract layer-by-layer coordinate distributions of optical anisotropy parameters (26)–(33) in blood film samples. The selection of phase planes $\theta _i$ in the object field of complex amplitudes, along with their corresponding physical depths $h_i$ (22) and effective depths $h_i^*$ (23) in our samples, is guided by optical-geometric approximations. Specifically, we consider $\Delta n\sim 10^{-3}$ and wavelength $\lambda =0.63~\upmu {\rm m}$ ^53–55^.

Utilizing (9) and (22), we estimate the phase intervals for scattering of various multiplicities in the object plane. A single pass of laser radiation through the polycrystalline blood film corresponds to the value $\theta _1\approx 0.7\,{\rm rad}\Leftrightarrow z_1\approx 70\,\upmu {\rm m}$. Similarly, double and triple passes correspond to $\theta _2\approx 1.4\,{\rm rad}\Leftrightarrow z_2^*\approx 140\,\upmu {\rm m}$ and $\theta _3\approx 2.1\,{\rm rad}\Leftrightarrow z_2^*\approx 210\,\upmu {\rm m}$, respectively.

In other words, phase shifts $\theta \le 0.7$ rad predominantly represent single scattering, while shifts in the range $0.7\,{\rm rad}\le \theta \le 1.4$ rad indicate low multiplicity scattering. For $\theta \ge 1.4$ rad, multiple scattering prevails. Performing scanning in the range of phase shifts $0.15\,{\rm rad}\le \theta \le 0.7$ rad enables a significant reduction in the influence of depolarized background and enhances the signal for $1\,\upmu {\rm m}\le h\le 70\,\upmu {\rm m}$. It’s important to note that this evaluation doesn’t account for the scattering multiplicity of optically active shaped elements at all depths in the polycrystalline blood film. Thus, we choose three phase planes corresponding to three regimes of laser light interaction with inhomogeneities in the blood film:

1. $\theta =0.2$ rad—Characterized by single scattering at both fibrillar networks of proteins (albumin, elastin, fibrin) and optically active shaped elements (erythrocytes, monocytes, leukocytes) in blood.
2. $\theta =0.6$ rad—Predominantly single scattering at fibrillar networks of proteins with an increased scattering multiplicity at optically active shaped elements in blood.
3. $\theta =1.0$ rad—Mainly associated with multiple scattering at optically active shaped elements in blood.

**Figure 3:**
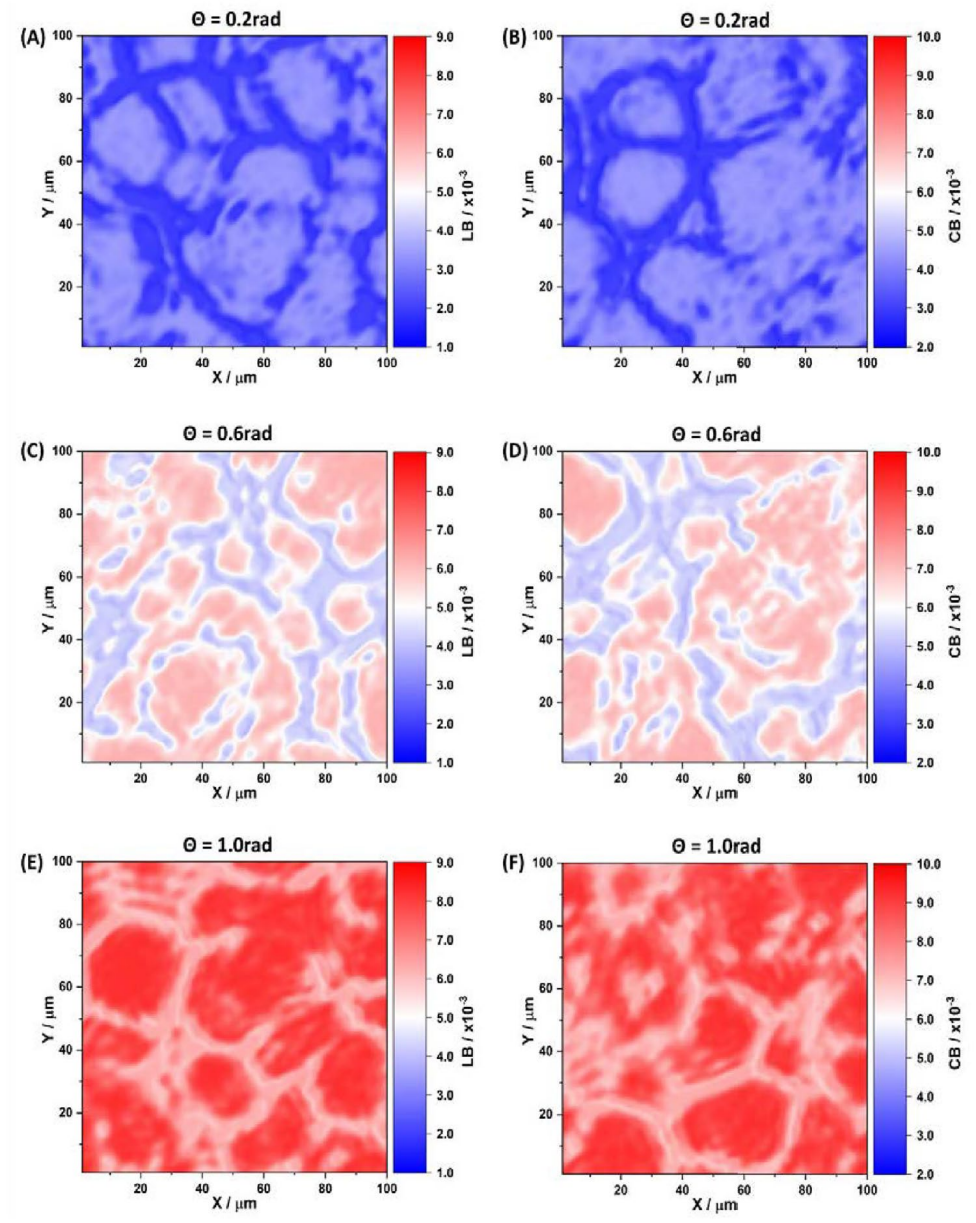
Maps of the linear $LB\left( \theta _k,m,n\right)$ (**A,C,E**) and circular $CB\left( \theta _k,m,n\right)$ birefringence (**B,D,F**) of a polycrystalline film of the blood of a healthy donor at “phase” sections of 0.2 rad (**A-B**), 0.6 rad (**C-D**) and 1.0 rad (**E-F**).

**Figure 4:**
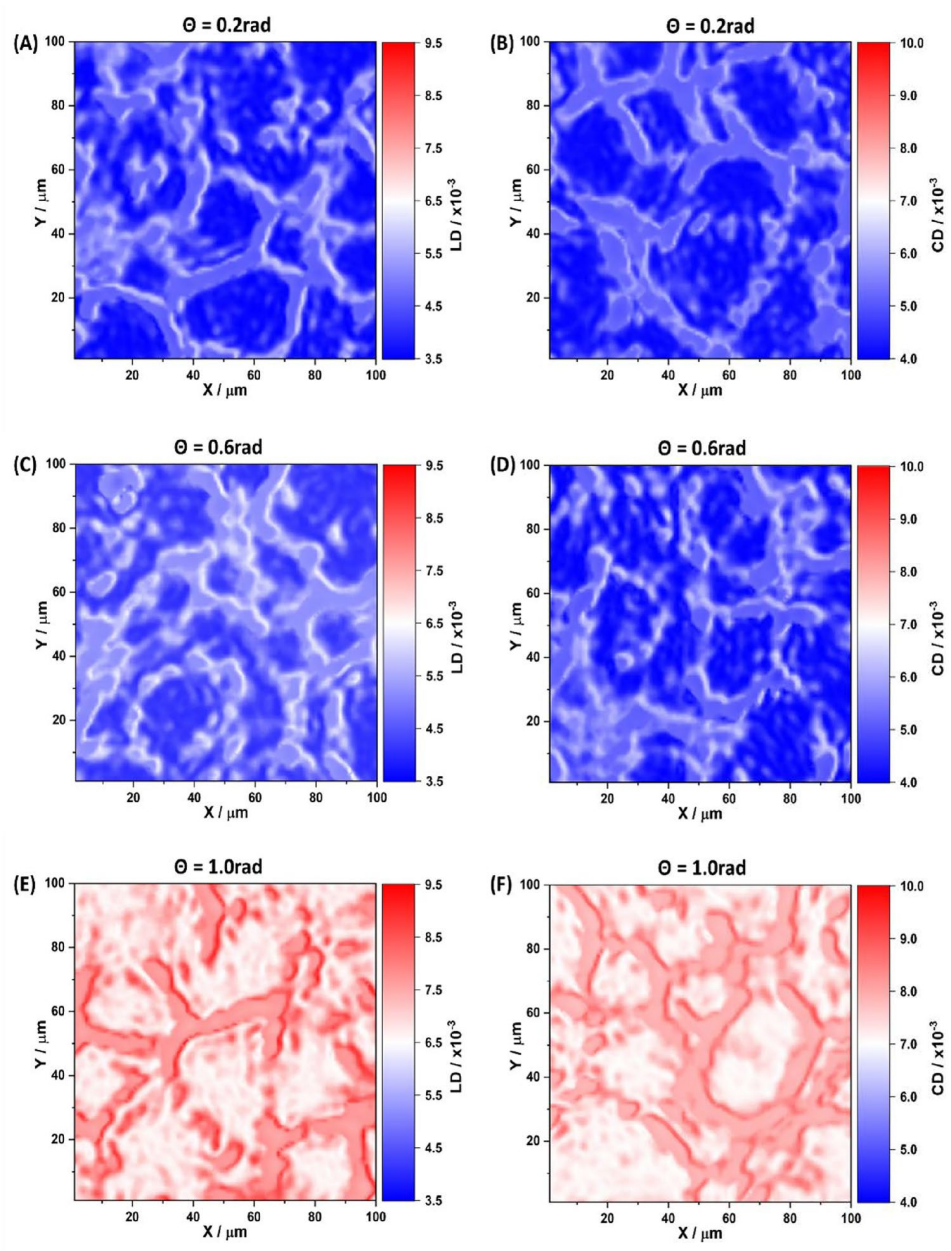
Maps of the linear $LD\left( \theta _k,m,n\right)$ (**A,C,E**) and circular $CD\left( \theta _k,m,n\right)$ dichroism (**B,D,F**) of a polycrystalline film of the blood of a healthy donor at “phase” sections of 0.2 rad (**A-B**), 0.6 rad (**C-D**) and 1.0 rad (**E-F**).

Figures 3 and 4 depict maps illustrating the phase and amplitude anisotropies of the blood film for a series of phase planes $\theta _k=0.2$ rad; 0.6 rad; 1.0 rad. The analysis of the layer-by-layer maps of the phase (Figure 3) and amplitude (Figure 4) anisotropies of the polycrystalline blood film reveals several key findings. Firstly, all types of optical anisotropy, denoted as *G* and including linear birefringence (*LB*), circular birefringence ($\left\langle CB\right\rangle$), linear dichroism (*LD*), and circular dichroism ($\left\langle CD\right\rangle$), are present in the polycrystalline structure of the blood film. This observation indicates the existence of supramolecular structural anisotropy, specifically *LB* and *LD*, formed by the polycrystalline networks of protein molecules. It also suggests that optically active shaped elements of blood contribute to the formation of circular birefringence ($\left\langle CB\right\rangle$) and dichroism ($\left\langle CD\right\rangle$). Furthermore, the individual topological structure (*m*, *n*) of optical anisotropy maps (*G*) can be discerned at each phase section ($\theta$) of the blood film object field. The coordinate heterogeneity of $G\left( m,n\right)$ distributions can be explained by the specificity of processes involving supramolecular spatial-angular crystallization of protein molecules and blood film dehydration. Lastly, the average level and range of optical anisotropy parameters ($G\equiv LB,\left\langle CB\right\rangle ,LD,\left\langle CD\right\rangle$) exhibit an increase with the increment of physical $h_i$ and effective $h_i^*$ depths in the polycrystalline blood film. This is attributed to the fact that the increase in physical depth ($\theta \le 0.7$ rad) corresponds to an enhancement in the degree of spatial-angular ordering of supramolecular protein networks ($LB\uparrow ,LD\uparrow$) and the number of formed elements ($\left\langle C B\right\rangle \uparrow ,\left\langle C D\right\rangle \uparrow$) in the polycrystalline blood film. Within the range of multiple scattering ($0.7\,{\rm rad}\le \theta \le 1.4$ rad), this process is intensified for $h_i^*$.

**Figure 5:**
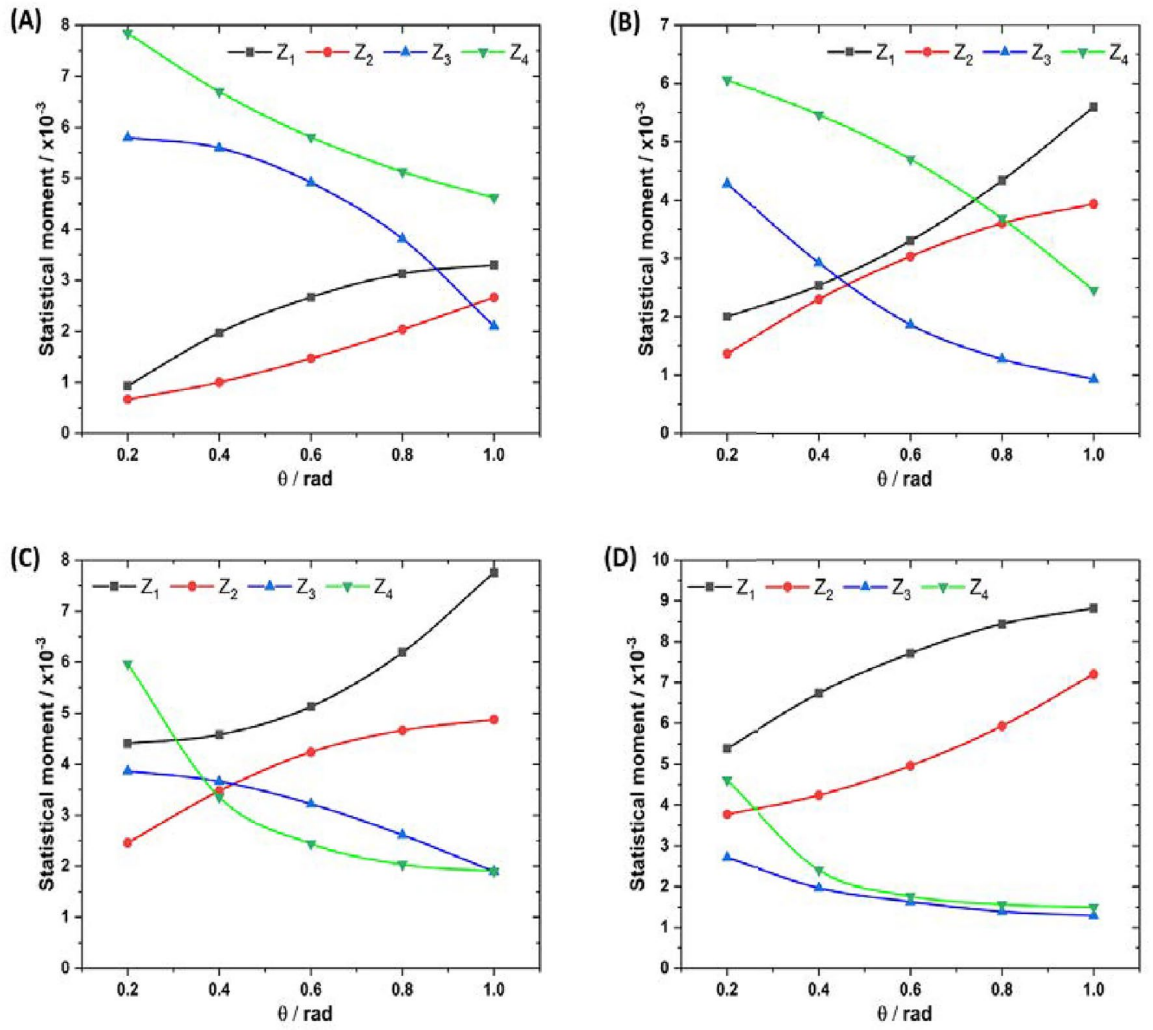
Phase-dependent magnitudes of the 1st (black, squares—$\times 10^{-3}$), 2nd (red, circles—$\times 10^{-3}$), 3rd (blue, upwards triangles), and 4th (green, downwards triangles) statistical moments characterizing the distributions of (**A**) linear birefringence, (**B**) circular birefringence, (**C**) linear dichroism, and (**D**) circular dichroism in a polycrystalline blood film from a healthy donor.

To quantitatively assess the transformation dynamics of algorithmically reconstructed optical anisotropy maps ($G\equiv LB,\left\langle C B\right\rangle ,LD,\left\langle C D\right\rangle$) at each phase plane $\theta _k$, a statistical analysis is conducted according to (34). Figure 5 illustrates a series of “phase” dependencies for the magnitudes of the 1st to 4th orders statistical moments ($Z_{i=1;2;3;4}\left( \theta \right)$). The analysis of the obtained data revealed two contrasting scenarios for the behavior of $Z_{i=1;2;3;4}\left( \theta \right)$. The first scenario involves a monotonic ”phase” increase in the 1st and 2nd statistical moments, which characterize the mean and variance of the $G\left( \theta ,m,n\right)$ distribution. The second scenario entails a decrement in the 3rd and 4th statistical moments, which characterize the skewness and kurtosis of optical anisotropy parameters. This behavior is attributed to the increased scattering multiplicity at $h_i^*(\theta \ge 0.7$ rad).

A multitude of optically anisotropic domains collectively contribute to the establishment of the average level of phase and amplitude anisotropy. Simultaneously, diverse geometric and concentration parameters within the fibrillar networks of proteins and optically active shaped elements of blood lead to an augmentation in the dispersion of linear and circular birefringence and dichroism in the polycrystalline blood film. The quantitative impacts of these processes are reflected in the values of the statistical moments. In the limit case, in accordance with the central limit theorem, the distribution $G\left( \theta \uparrow \right) \equiv LB,\left\langle C B\right\rangle ,LD,\left\langle C D\right\rangle$ tends toward the normal distribution, and $Z_{3;4}\rightarrow 0$.

Comparing the changes in the 1st to 4th orders statistical moments, it was observed that skewness ($Z_3$) and kurtosis ($Z_4$) exhibit greater sensitivity to phase changes in the distributions of linear and circular birefringence and dichroism $G\left( \theta \right)$. This heightened sensitivity may be attributed to the fact that small variations in ($Z_2$) lead to larger changes in higher-order statistical moments. In the range $0.2\,{\rm rad}\le \theta \le 0.7$ rad, the dynamic range of ${\Delta }Z_{3;4}$ changes corresponding to linear birefringence and dichroism is 2.5–3 times, while for circular birefringence and dichroism, it is 3–4 times. Therefore, the layer-by-layer assessment of the polycrystalline structure in the phase shift range $0.2\,{\rm rad}\le \theta \le 0.7$ rad holds the potential for early-stage detection of oncological changes in the optical anisotropy of fibrillar networks of proteins and optically active shaped elements.

## Discussion

### Polycrystalline blood film diagnosis

**Figure 6:**
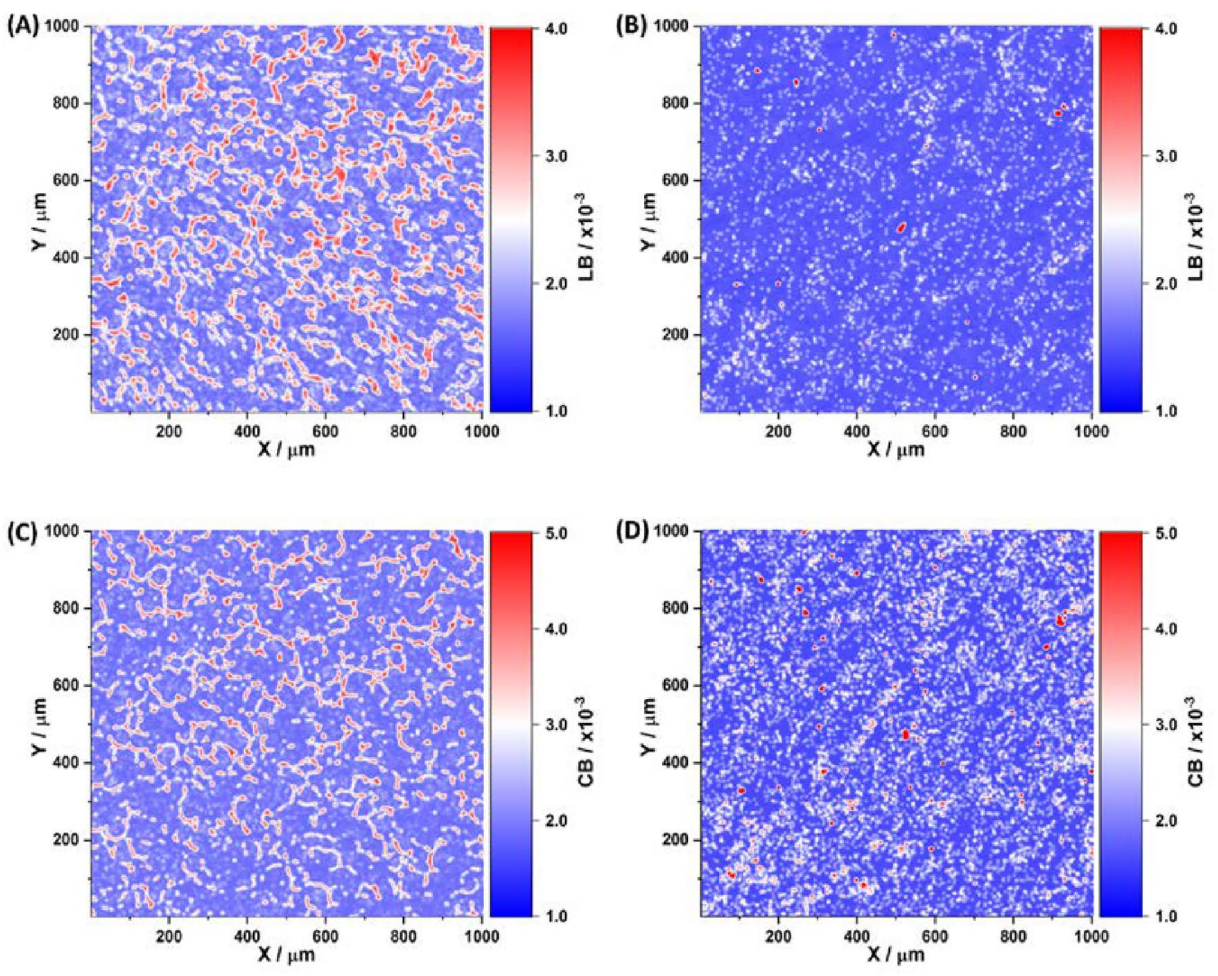
Maps of the (**A-B**) linear $LB(\theta ^*=0.45\,{\rm rad},m,n)$ and (**C-D**) circular $CB(\theta ^*=0.65\,{\rm rad},m,n)$ birefringence of polycrystalline blood films obtained for group 1 (**A,C**) and group 2 (**B,D**).

The optimal phase planes for diagnostic purpose have been unidentified: $\theta ^*( LB,\left\langle CB\right\rangle )=0.65$ rad and $\theta ^*(LB,\left\langle CB\right\rangle )=0.45$ rad. The optical anisotropy parameters obtained in these planes are illustrated in Figs. 5 and 6 for samples from group 1 (healthy) and group 2 (moderately differentiated prostate adenocarcinoma with a Gleason’s pattern scale of $3+3$).

#### Quantitative evaluation of polarization maps

To assess the layer-by-layer maps of optical anisotropy (*G*), statistical moments of the first ($Z_1$), second ($Z_2$), third ($Z_3$), and fourth ($Z_4$) orders are utilized and calculated as follows ^53,56,57^:

$$\begin{aligned} \begin{array}{lrlr} \displaystyle Z_1 = {\frac{1}{P}} \sum _{j=1}^{P} (G(\theta ,m\times n))_j;\\ \displaystyle Z_2=\sqrt{\frac{1}{P}\sum _{j=1}^{P} (G^2(\theta ,m\times n))_j};\\ \displaystyle Z_3=\frac{1}{Z_2^3}\frac{1}{P}\sum _{j=1}^{P} (G^3(\theta ,m\times n))_j;\\ \displaystyle Z_4=\frac{1}{Z_2^4}\frac{1}{P}\sum _{j=1}^{P} (G^43(\theta ,m\times n))_j,\\ \end{array}. \end{aligned}$$

where $P=m\times n$ is the *xy* resolution of the camera. These measures ($Z_1$–$Z_4$) most fully characterize the mean, variance, skewness, and kurtosis of the layer-by-layer ($\theta _k$) distributions $G(\theta _k,m,n)$ of linear and circular birefringence ($LB, \langle CB \rangle$) and dichroism ($LD, \langle CD \rangle$).

The methodology for implementing this statistical approach involves several steps. Initially, groups of blood polycrystalline film samples are formed from both healthy and diseased patients. For each sample within each group, anisotropy maps, denoted as $G(\theta _k,m,n)$, are obtained for a series (*H*) of phase sections $\theta _{(k=1...H)}$. Subsequently, a set of statistical moments of 1st to 4th orders, denoted as $Z_{i=1-4}$, is calculated using (35).

Within each group, the mean value $\bar{Z}{i=1-4}$ and standard deviation $\sigma {i=1-4}$ are computed for the obtained distribution of $Z_{i=1-4}$. Further, statistically significant ($p\le 0.05$) intergroup differences (“norma”—“cancer”—“stage”) for each phase section $\theta _{k=1...H}$ are determined for each statistical moment of 1st to 4th orders $Z_{i=1-4}$ ($LB,\langle CB \rangle ,LD,\langle CD\rangle$). The measures $Z_{i=1-4}^*$ ($LB,\langle CB \rangle ,LD,\langle CD\rangle$) are then employed in algorithms for the information analysis of evidence-based medicine ^58–60^.

#### Quantitative analysis of anisotropy maps

For each statistically significant parameter $Z_{i=1-4}^*$ ($LB,\langle CB \rangle ,LD,\langle CD\rangle$) the criteria of evidence-based medicine has been used ^58–60^:

1. Sensitivity (*Se*) - proportion of true positive results (*TP*) among the group of diseased ($D_+$) patients
  40 $$\begin{aligned} Se=\frac{TP}{D_+}100\%, \end{aligned}$$
2. Specificity (*Sp*) - proportion of true negative results (*TN*) among the control group of healthy patients ($D_-$)
  41 $$\begin{aligned} Sp=\frac{TP}{D_-}100\%, \end{aligned}$$
3. Accuracy (*Ac*) - proportion of true results ($TP+TN$) among all the patients ($D_++D_-$)
  42 $$\begin{aligned} Ac=\frac{TP+TN}{D_++D_-}100\%. \end{aligned}$$
  In our study, accuracy refers to the quantity of accurate diagnoses achieved through the utilization of 3D layer-by-layer MM reconstruction for anisotropy mapping of the polycrystalline structure in blood films.

In samples from group 2, a decrease in both the average level and fluctuations of linear birefringence and dichroism was observed. Conversely, an increase in both the average level and fluctuations of circular birefringence and dichroism was noted in the same group. From a physical perspective, these results can be linked to changes in the ratio between the concentrations of albumin and blood globulin proteins. It is well-documented ^30–33,61^ that early malignant processes are accompanied by an elevation in the concentration of optically active globulin molecules. The increased globulin concentration in group 2 contributes to the heightened magnitude of circular birefringence and dichroism compared to the healthy group.

The reduction in the concentration of albumin molecules, in turn, leads to a decrease in the level of linear birefringence and dichroism of supramolecular protein networks. These biological changes are reflected in the intergroup differences $\Delta Z_i$ of the statistical moments $Z_i$ characterizing the optical anisotropy maps of polycrystalline blood films from groups 1 and 2 (Table 2).

**Table 2:** Intergroup statistical parameters of anisotropy parameters of the control and study groups of blood facies samples.

Groups	Group 1–Group 2			
$\Delta Z_i=1;2;3;4$	*LB*	$\left\langle CB\right\rangle$	*LD*	$\left\langle CD\right\rangle$
$\Delta Z_1$	$0.06\pm 0.002$	$0.09\pm 0.004$	$0.05\pm 0.002$	$0.08\pm 0.003$
$\Delta Z_2$	$0.045\pm 0.002$	$0.075\pm 0.003$	$0.036\pm 0.002$	$0.064\pm 0.003$
$\Delta Z_3$	$0.28\pm 0.012$	$0.35\pm 0.014$	$0.33\pm 0.013$	$0.49\pm 0.018$
$\Delta Z_4$	$0.37\pm 0.015$	$0.52\pm 0.023$	$0.42\pm 0.017$	$0.063\pm 0.027$

Groups	Group 1–Group 3			
$\Delta Z_i=1;2;3;4$	*LB*	$\left\langle CB\right\rangle$	*LD*	$\left\langle CD\right\rangle$
$\Delta Z_1$	$0.09\pm 0.005$	$0.12\pm 0.0007$	$0.075\pm 0.0005$	$0.105\pm 0.006$
$\Delta Z_2$	$0.063\pm 0.004$	$0.087\pm 0.0005$	$0.054\pm 0.0003$	$0.079\pm 0.0005$
$\Delta Z_3$	$0.37\pm 0.019$	$0.44\pm 0.025$	$0.41\pm 0.024$	$0.54\pm 0.029$
$\Delta Z_4$	$0.48\pm 0.026$	$0.53\pm 0.027$	$0.52\pm 0.027$	$0.061\pm 0.033$

Groups	Group 2–Group 3			
$\Delta Z_i=1;2;3;4$	*LB*	$\left\langle CB\right\rangle$	*LD*	$\left\langle CD\right\rangle$
$\Delta Z_1$	$0.04\pm 0.003$	$0.07\pm 0.004$	$0.03\pm 0.002$	$0.05\pm 0.003$
$\Delta Z_2$	$0.03\pm 0.002$	$0.05\pm 0.003$	$0.024\pm 0.001$	$0.03\pm 0.0003$
$\Delta Z_3$	$0.19\pm 0.011$	$0.27\pm 0.015$	$0.24\pm 0.014$	$0.34\pm 0.019$
$\Delta Z_4$	$0.28\pm 0.016$	$0.39\pm 0.021$	$0.36\pm 0.019$	$0.48\pm 0.026$

The 4th-order statistical moment, representing the kurtosis of the distributions of phase ($LB, \langle CB\rangle$) and amplitude ($LD, \langle CD\rangle$) anisotropy parameters in polycrystalline blood films, demonstrates remarkable sensitivity to early signs of an oncological state.

Table 3 presents the sensitivity, specificity (*Sp*), and balanced accuracy (*Ac*) values for the early diagnosis of prostate cancer using the 3D layer-by-layer MM mapping method. These values are calculated based on the intergroup difference in the fourth-order statistical moment for each of the four optical anisotropy parameters. The results reveal an excellent level of balanced accuracy, indicating high levels of selectivity and specificity in the diagnostic approach.

**Table 3:** Operational characteristics of intergroup differential diagnosis of blood samples.

Groups	Group 1–Group 2			
Parameters	*LB*	$\left\langle CB\right\rangle$	*LD*	$\left\langle CD\right\rangle$
$Se, \%$	86.1	94.4	88.9	97.2
$Sp, \%$	83.3	91.7	86.1	94.4
$Ac, \%$	84.7	93.1	87.5	95.8

Groups	Group 1–Group 3			
Parameters	*LB*	$\left\langle CB\right\rangle$	*LD*	$\left\langle CD\right\rangle$
$Se, \%$	93.1	95.9	90.3	98.6
$Sp, \%$	90.3	91.7	88.9	95.9
$Ac, \%$	91.7	93.8	89.6	97.3

Groups	Group 2–Group 3			
Parameters	*LB*	$\left\langle CB\right\rangle$	*LD*	$\left\langle CD\right\rangle$
$Se, \%$	90.3	91.7	88.9	93.1
$Sp, \%$	88.9	90.3	87.5	91.7
$Ac, \%$	89.6	91.0	88.2	92.4

A comparative analysis of diagnostic efficacy was conducted with three existing polarimetric methods, as outlined in Table 4. The considered methods are:

i. Azimuth-invariant polarization mapping of the distributions of polarization azimuth $\alpha (m,n)$ in the object field of the biological layer ^30–33,62–64^;
ii. Azimuth-invariant polarization mapping of the distributions of polarization ellipticity $\beta (m,n)$ in the object field of the biological layer ^30,48,49,53–56^;
iii. MM ($R_{ik}(m,n)$) mapping of biological layers ^30,61,65^;
iv. This work: 3D MM reconstruction ($3D-LB, \left\langle CB\right\rangle , LD, \left\langle CD\right\rangle$) of the parameters of phase and amplitude anisotropy in biological layers in 3D.

An assessment of the diagnostic effectiveness of 2D and 3D polarization mapping methods for prostate tumor layers with varying optical thickness revealed that, for partially depolarizing polycrystalline blood films ($\Lambda =40-45\%$), the balanced accuracy of coordinate polarization methods ($\alpha , \beta (m,n)$) and MM mapping mostly falls below a satisfactory level. However, the accuracy of early differential diagnosis achieved through the 3D MM reconstruction method described in this work represents a significant improvement.

**Table 4:** Balanced accuracy of different laser polarimetry methods for differentiating partially depolarising ($\Lambda =40\%-45\%$) polycrystalline blood films from healthy donors and patients with highly differentiated adenocarcinoma.

	$\alpha (m,n)$	$\beta (m,n)$
$Ac, \%$	55–65	60–65
	$R_{ik}(m,n)$	$3D-LB,\left\langle CB\right\rangle ,LD,\left\langle CD\right\rangle$ (This work)
$Ac, \%$	70–75	93–95

### From bench to bedside: envisioning the clinical role of MM mapping

The presented analysis includes sensitivity (*Se*), specificity (*Sp*), and balanced accuracy (*Ac*) for the comparison of group 2 (moderately differentiated prostate adenocarcinoma, $3+3$ on Gleason’s pattern scale) and group 3 (poorly differentiated prostate adenocarcinoma, $4+4$ on Gleason’s Pattern scale) (see Table 5).

The results in Table 5 indicate a high level of efficiency (ranging from $90.3\%$ to $95.8\%$) in diagnosing prostate tumors through MM mapping of polycrystalline blood films from patients with prostate adenocarcinoma at varying degrees of differentiation.

**Table 5:** Operational characteristics of the diagnostic power of the 3D MM method for prostate adenocarcinoma stage differentiation.

Operational characteristic	$\theta ^*=0.65$ rad		$\theta ^*=0.45$ rad	
	*LB*	$\left\langle CB\right\rangle$	*LD*	$\left\langle CD\right\rangle$
$Se, \%$	94.4	97.2	91.7	94.4
$Sp, \%$	91.7	94.4	88.9	94.4
$Ac, \%$	93.1	95.8	90.3	94.4

In conclusion, our study employed a 3D MM reconstruction approach for multiparameter polarimetry studies on the polycrystalline structure of dehydrated blood smears. The investigation revealed method’s sensitivity to subtle changes in optical anisotropy properties resulting from alterations in the quaternary and tertiary structures of blood proteins, leading to disturbances in crystallization structures at the macro level at the very early stage of a disease. More specifically, the developed 3D MM diagnostic approach demonstrated discernible early cancer-related alterations in optical anisotropy properties. This included an examination of spatial distributions of linear and circular birefringence and dichroism in partially depolarizing polycrystalline blood films sourced from healthy tissues and cancerous prostate tissues across various stages of adenocarcinoma. Observable and quantifiable changes in the 1st to 4th order statistical moments, characterizing the distributions of optical anisotropy parameters, were identified in different “phase” sections of the blood smear volumes.

Emphasizing the advantages of the presented diagnostic approach over traditional methods, we highlighted its cost-effectiveness and simplicity, requiring only a basic polarization-based optical setup without the need for reagents. Additionally, the analysis of dehydrated blood samples is prompt, providing express results compared to the time-consuming nature of biochemical analysis. Notably, during measurements, all parameters of the polycrystalline structure can be assessed simultaneously. An excellent accuracy ($> 90\%$) for early cancer diagnosis and differentiation of its stages is achieved, demonstrating the technique’s significant potential for rapid and accurate definitive cancer diagnosis compared to existing screening approaches. This pioneering work marks an initial step toward the development of an advanced, practical, and cost-effective toolkit for expedited, minimally invasive cancer diagnosis, integrated with conventional blood tests.

## Methods and materials

### 3D Mueller matrix imaging approach

**Figure 7:**
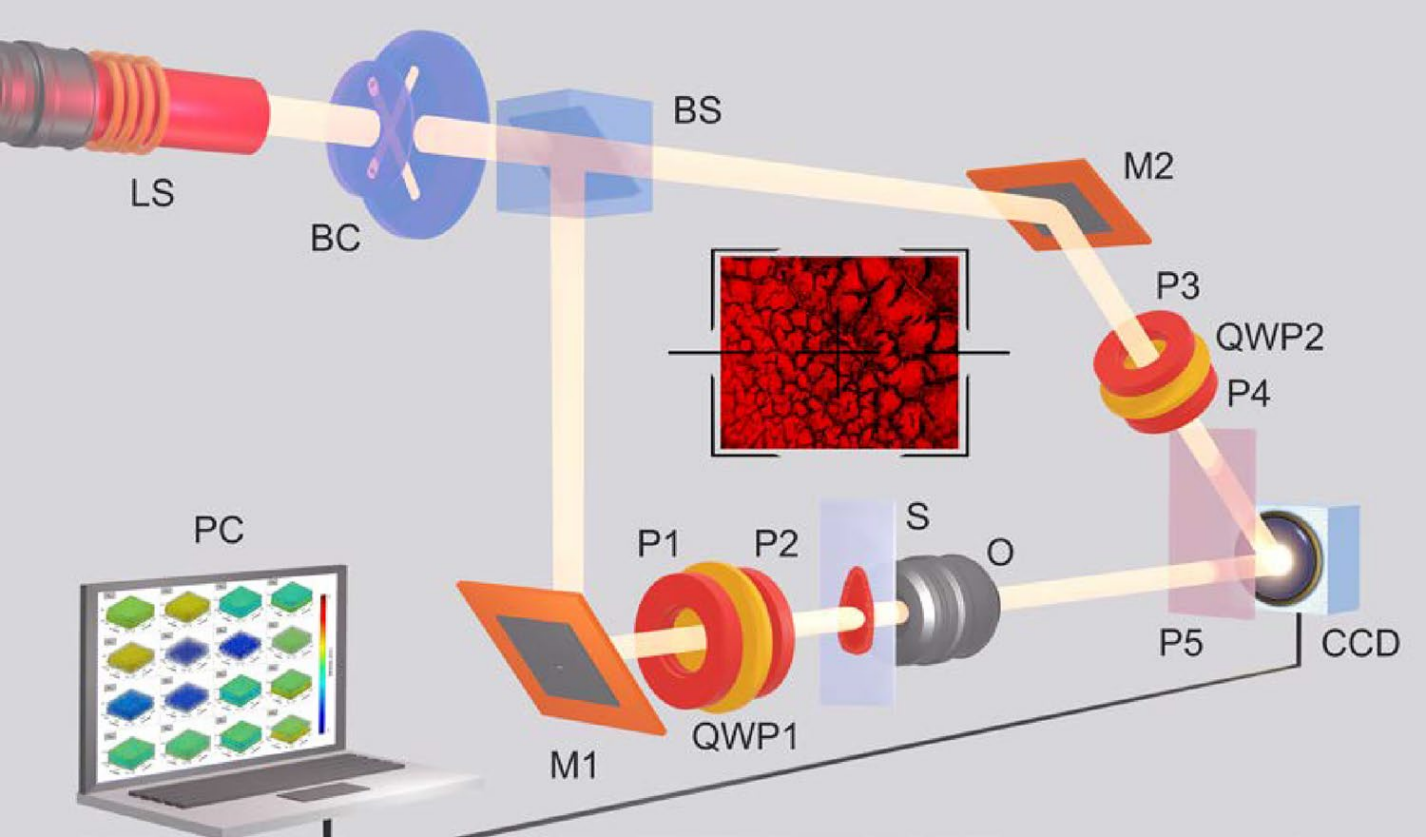
The optical scheme used for MM imaging approach: LS—He–Ne laser; BC—collimator; BS—50–50 beam splitter; M1,M2—rotating mirrors; P1,P2,P3,P4—linear polarisers; QWP1, QWP2—quarter wave plate; S—the polycrystalline blood film sample under investigation; O—objective; P5—linear polariser (analyser); CCD—digital camera; PC—personal computer. See also Supplementary video 1.

Figure 7 shows a schematic of the 3D MM imaging experimental setup used in the studies of blood films (see also Supplementary video 1).

A parallel (2000 µm in diameter) beam of He-Ne (633 nm) laser is collimated before being split into equal ”irradiating” and “reference” beams. Each beam passed through an equivalent polarising filter set to control the polarisation. The irradiating beam passed through the sample, and the image is projected by an objective, through a polariser, into the imaging plane of the digital camera. The reference beam is also guided into the imaging plane of the camera, and an interference pattern is formed from the superposition of the two beams. The camera records the intensity distribution of the interference pattern, which is then computationally analysed.

The layer-by-layer 3D MM polarimetry setup was calibrated using model birefringent phase-shifting plates, including $1/4\lambda$ and $1/2\lambda$ configurations. The accuracy of measuring the magnitude of the MM elements is: $R_{i,j} \sim 0.005$ for $i=1;2;3;j=1;2;3$, and $R_{i,j} \sim 0.01$ for $i=1;2;3;4;j=4;$ and $i=4;j=1;2;3;4$.

The design of the polarization interferometer establishes a foundational aperture angle of convergence for the reference and object beams at $\Phi _0=0.25$ rad. This setup includes mirrors *M*1 and *M*2, which are spaced $D=20$ cm apart, with the interference pattern’s registration plane located at a distance of $L=60$ cm. Additionally, the aperture angle $\Phi _0$ is adjustable, taking into account the angular field of view of the polarizing microlens *O*, which permits variations in $\Phi _0$ from a minimum of $\Phi _{min}=0.12$ rad to a maximum of $\Phi _{max}=0.23$ rad, with an adjustment capability of ${\Delta \phi }=0.1$ rad. As a result, in the plane of the photosensitive area CCD (The Imaging Source DMK 41AU02. AS, monochrome 1/2 ”CCD, Sony ICX205AL (progressive scan) resolution—$1280\times 960$, size of the photosensitive area—$7600\times 6200~\upmu {\rm m}$; sensitivity—0.05 *lx*; dynamic range—8 bit, SNR—9 bit), the photosensitive area of which contains $m\times n=1280\times 960$ pixels) bands of interference patterns with a width of $B_{min}\approx 3~\upmu {\rm m};\ B_0\approx 4~\upmu {\rm m}\ {\text {and}}\ B_{max}\approx 5~\upmu {\rm m}$ are formed. Thus, within the limits of changing the aperture angle in the plane of the digital camera, $N=2000, 1500$ and 1200 interference fringes are recorded, respectively. In accordance with this, $k=5$; 4 and 3 pixels take part in the detection of each local band. Each pixel has a geometric resolution of 3 µm. In other words, the interference polarimeter provides resolution of the structural elements of the blood facies in the range from 3 µm to 3600 µm–6000 µm. This level is comparable to the minimal geometric dimensions of albumin-globulin molecules and their optically anisotropic supramolecular network maximal dimensions. Promising in terms of optimization of installation parameters in order to obtain lower frequency information about the structure of interference distributions, it is necessary to reduce the value of the base aperture angle $\Phi _0$. This is problematic in this experimental setup. Therefore, it is necessary to use a different interference scheme. To expand the range of registration of geometric dimensions and study large-scale fibrillar networks of native histological sections of prostate tumor biopsies, it is advisable to use a Mach-Zehnder polarization interferometer circuit. In this situation, variations in the base aperture angle $\Phi _0$ are determined by the angular field of view of the polarizing microlens *O* (${\Delta \phi }=0.1$ rad) and make up the range—$\Phi _0=0$ rad; $\Phi _{min;max}=0.05$ rad. As a result, significantly wider (${B\gg B}_{min}=10$ µm) interference fringes are recorded by a set of pixels of a digital CCD camera. The total time for image acquisition, its reconstruction and algorithmic processing does not exceed 2–3 min. The performance of such a system can be significantly increased by using, instead of classical polarization elements, electro-optical polarization modulators and pixelated polarization camera ^66,67^ In the future, the compact overall dimensions of the Mach-Zehnder polarization interferometer can be reduced to tens of centimeters (prototype $245\times 330\times 25$ mm, 3B Scientific, U10353 [1014617]). We wait that the total time for experimental measurements will be some seconds.

The showcased setup functions as a laboratory prototype of a 3D MM tomography tailored for layer-by-layer imaging of the polycrystalline structure in biological tissues and fluids from human organs. Future enhancements are targeted at automating the optical and polarization elements, fine-tuning reconstruction algorithms, and obtaining 3D distributions of anisotropy parameters with the ultimate goal to amalgamate the principles of 3D MM reconstruction with fiber-optic systems, thereby extending the methodology for measuring optical anisotropy parameters in vivo.

The presented experimental setup serves as a laboratory prototype for a 3D MM tomography designed specifically for the layer-by-layer imaging of the polycrystalline structure in biological tissues and fluids from human organs ^10,38^. Future developments are aimed at automating both the optical and polarization components, refining the reconstruction algorithms, and achieving 3D distributions of anisotropy parameters with the ultimate objective to extend the methodology for robotic automatic standalone optical biopsy and definitive histopathology diagnostics.

### Samples of blood films

In the current study, blood smears are considered as a primary example of evaporated biological liquids. These thin (2–5 µm) blood films exhibit a heterogeneous, complex polycrystalline structure (see Fig. 1) characterized by varying light scattering multiplicities and distinctive depolarizing capabilities.

In essence, a biological fluid film represents a spatially inhomogeneous and optically anisotropic structure, comprised of various biochemical and molecular crystalline complexes. This film contains elements characterized by multiple optical scattering, including erythrocytes, platelets, and leukocytes, which exhibit circular birefringence and dichroism ^68^.

For the experiment, polycrystalline blood film samples were collected from both healthy and diseased volunteers. The blood film samples were prepared by applying a blood drop to an optically homogeneous cover glass heated up to $36.6^{\circ }$ in advance. The blood drops fully dehydrated within 40–45 min.

Three distinct groups of blood film samples were created:

Group 1 included $k=36$ samples from healthy volunteers.

Group 2 included $k=36$ samples from volunteers diagnosed with moderately differentiated prostate adenocarcinoma ($3+3$ on Gleason’s pattern scale).

Group 3 included $k=36$ samples from volunteers diagnosed with poorly differentiated prostate adenocarcinoma ($4+4$ on Gleason’s Pattern scale).

Table 6. Optical properties of polycrystalline blood film samples for the groups.

**Table 6:** Optical parameters of polycrystalline blood film samples.

Parameter	Group 1	Group 2	Group 3
Attenuation (extinction)	$0.64 \pm$	$0.66 \pm$	$0.62 \pm$
coefficient $\tau , {\text {cm}}^{-1}$	$\pm 0.035$	$\pm 0.039$	$\pm 0.032$
Depolarisation degree	$39 \pm$	$44 \pm$	$42 \pm$
$\Lambda ,\%$	$\pm 0.77$	$\pm 0.084$	$\pm 0.81$

The extinction coefficient ($\tau , {\text {cm}}^{-1}$) of polycrystalline blood film samples is determined following the established photometric method, measuring the attenuation of illuminating beam intensity by the sample ^69^. This process utilized an integral light-scattering sphere ^70^. Additionally, the integral degree of depolarization ($\Lambda , \%$) for polycrystalline blood film samples is assessed utilizing standard MM polarimetry ^53,56,57^.

To determine the statistical significance of a representative sampling of the number of samples by the cross-validation method ^71^, the standard deviation $\sigma ^2$ of each of the calculated values of the statistical moments $Z_{i=1;2;3;4}(k)$ is determined. The specified number (36 for each group) of samples provided the level $\sigma ^2\le 0.025$. This standard deviation corresponds to a confidence interval $p<0.05$, demonstrating the statistical reliability of the 3D MM mapping method.

The sample preparation procedure adhered to the principles of the Declaration of Helsinki and complied with the International Conference on Harmonization-Good Clinical Practice and local regulatory requirements. The study received review and approval from the appropriate Independent Ethics Committees, and written informed consent is obtained from all subjects prior to study initiation.

### Diagnostic algorithmic framework

An analytical protocol for distinguishing between normal (healthy) and abnormal (e.g., cancerous prostate) tissues is outlined as follows. Initially, the identification of the phase plane, denoted as $\theta ^*$, which demonstrates heightened sensitivity to pathological alterations in the optical anisotropy parameters of the polycrystalline blood structure, is undertaken as:

1. An initial “macro” phase scanning step $\theta _k^{max}=0.05, {\text {rad}}$ is selected.
2. The layer-by-layer coordinate distributions $G(\theta _k,m,n)$ are reconstructed for each $\theta _k^{max}$.
3. The statistical moments $Z_{i=1;2;3;4}$ are calculated.
4. The differences between the values of each of the statistical moments are calculated $(\Delta Z_i)k=Z_i(\theta {j+1}^{max})-Z_i(\theta _{j}^{max})$.
5. The phase interval $\Delta \theta ^*=\theta _{j+1}^{max}-\theta _{j}^{max}$ within which the monotonic increase in the value of $(\Delta Z_i)k=Z_i(\theta {j+1}^{max})-Z_i(\theta _{j}^{max})$ stopped is determined.
6. Within the limits $\Delta \theta ^*$, a new series of values $(\Delta Z_i)k=Z_i(\theta {q+1}^{min})-Z_i(\theta _{q}^{min})$ is calculated with a discrete “micro” phase scanning step $\theta _q^{min}=0.01$ rad.
7. For each optical anisotropy parameter in *G*, the optimal phase plane $\theta ^*$, in which $\Delta Z_i(\theta ^*)=max$, is determined.
8. In these planes ($\theta ^*$ ), the mean $\bar{Z}_{i=1;2;3;4}^*$ and standard deviations $\sigma (\Delta Z_i^*)$ are determined for comparing between groups 1 and 2, as well as for comparing between group 2 and 3. The sensitivity (*Se*), specificity (*Sp*) and balanced accuracy (*Ac*) are also calculated ^58–60,71^.

### Ethics approval

This study was conducted in accordance with the principles of the Declaration of Helsinki, and in compliance with the International Conference on Harmonization-Good Clinical Practice and local regulatory requirements. Ethical approval was obtained from the Ethics Committee of the Bureau of Forensic Medicine of the Bukovinian State Medical University (Protocol No. 8 dated May 18, 2023), Chernivtsi, Ukraine.

### Supplementary Information

Supplementary Video 1.

## Data Availability

The datasets and code used during the current study available from the corresponding author on reasonable request.
