# Author Correction: Insights into polycrystalline microstructure of blood films with 3D Mueller matrix imaging approach

**DOI:** 10.1038/s41598-024-69469-2

**Published:** 2024-08-13

**Authors:** Alexander G. Ushenko, Anton Sdobnov, Irina V. Soltys, Yuriy A. Ushenko, Alexander V. Dubolazov, Valery M. Sklyarchuk, Alexander V. Olar, Liliya Trifonyuk, Alexander Doronin, Wenjun Yan, Alexander Bykov, Igor Meglinski

**Affiliations:** 1 Taizhou Institute of Zhejiang University, Taizhou, 310027 China; 2 Optics and Publishing Department, Yuriy Fedkovych Chernivtsi National University, 2 Kotsyubynskyi Str, Chernivtsi, 58002 Ukraine; 3 Optoelectronics and Measurement Techniques, University of Oulu, P.O. Box 4500, 900014 Oulu, Finland; 4 Department of Physics, Shaoxing University, Shaoxing, 312000 Zhejiang China; 5 Computer Science Department, Yuriy Fedkovych Chernivtsi National University, 2 Kotsyubynskyi Str., Chernivtsi, 58002 Ukraine; 6 Rivne State Medical Center, 78 Kyivska Str., Rivne, 33007 Ukraine; 7 School of Engineering and Computer Science, Victoria University of Wellington, 6140 Wellington, New Zealand; 8 College of Engineering and Physical Sciences, Aston University, Birmingham, B4 7ET UK

Correction to: *Scientific Reports* 10.1038/s41598-024-63816-z, published online 13 June 2024

The original version of this Article contained errors in the Affiliations.

Alexander G. Ushenko was incorrectly affiliated with ‘College of Electrical Engineering, Zhejiang University, Hangzhou, 310027, China’. The correct affiliations are listed below.

Taizhou Institute of Zhejiang University, Taizhou, 310027, China

Optics and Publishing Department, Yuriy Fedkovych Chernivtsi National University, 2 Kotsyubynskyi Str., Chernivtsi, 58002, Ukraine

In addition, Affiliations 4 and 5 were not listed in the correct order. The correct affiliations are listed below:

Affiliation 4:

Department of Physics, Shaoxing University, Shaoxing, Zhejiang, 312000, China

Affiliation 5:

Computer Science Department, Yuriy Fedkovych Chernivtsi National University, 2 Kotsyubynskyi Str., Chernivtsi, 58002, Ukraine

As a result, the Affiliations have been renumbered.

The original Article has been corrected.

